# The Role of The A2A Receptor in Cell Apoptosis Caused by MDMA

**Published:** 2012-12-12

**Authors:** Mansooreh Soleimani, Majid Katebi, Akram Alizadeh, Farzaneh Mohammadzadeh, Mehdi Mehdizadeh

**Affiliations:** 1. Cellular and Molecular Research Center, Tehran University of Medical Sciences, Tehran, Iran; 2. Persian Gulf Research Center for Stem Cell Therapy and Department of Anatomy, Hormozgan University of Medical Sciences, Bandar Abbas, Iran; 3. Department of Tissue Engineering, Tehran University of Medical Sciences, Tehran, Iran; 4. Department of Anatomy, Tehran University of Medical Sciences, Tehran, Iran

**Keywords:** Ecstasy or MDMA, Neurotoxicity, Adenosine Receptor, Agonist of A2A Receptor, Antagonist of A2A Receptor

## Abstract

**Objective:**

Ecstasy, also known as 3, 4-methylenedioxymethamphetamine (MDMA), is a psychoactive recreational hallucinogenic substance and a major worldwide recreational drug. There are neurotoxic effects observed in laboratory animals and humans following MDMA use. MDMA causes apoptosis in neurons of the central nervous system (CNS). Withdrawal signs are attenuated by treatment with the adenosine receptor (A2A receptor). This study reports the effects of glutamyl cysteine synthetase (GCS), as an A2A receptor agonist, and succinylcholine (SCH), as an A2A receptor antagonist, on Sprague Dawley rats, both in the presence and absence of MDMA.

**Materials and Methods:**

In this experimental study, we used seven groups of Sprague Dawley rats (200-250 g each). Each group was treated with daily intraperitoneal (IP) injections for a period of one week, as follows: i. MDMA (10 mg/kg); ii. GCS (0.3 mg/kg); iii. SCH (0.3 mg/kg); iv. GCS + SCH (0.3 mg/kg each); v. MDMA (10 mg/kg) + GCS (0.3 mg/kg); vi. MDMA (10 mg/kg) + SCH (0.3 mg/kg); and vi. normal saline (1 cc/kg) as the sham group. Bax (apoptotic protein) and Bcl-2 (anti-apoptotic protein) expressions were evaluated by striatum using RT-PCR and Western blot analysis.

**Results:**

There was a significant increase in Bax protein expression in the MDMA+SCH group and a significant decrease in Bcl-2 protein expression in the MDMA+SCH group (p<0.05).

**Conclusion:**

A2A receptors have a role in the apoptotic effects of MDMA via the Bax and Bcl-2 pathways. An agonist of this receptor (GCS) decreases the cytotoxcity of MDMA, while the antagonist of this receptor (SCH) increases its cytotoxcity.

## Introduction

Ecstasy (3, 4-methylenedioxymethamphetamine; MDMA) is a psychoactive, recreational, hallucinogenic drug abused worldwide. Several studies express concern that MDMA has the ability to induce neurotoxic effects both in laboratory animals and humans. Despite more than two decades of research, the neurotoxic mechanisms of MDMA are not clear. MDMA induces serotonergic terminal loss in rats and some mice strains, and broader neuronal degeneration throughout several brain areas such as the cortex, hippocampus, and striatum. In human ecstasy abusers, there is evidence for deficits in seronergic biochemical markers (1, 2).

MDMA causes hallucination and psycho-stimulation, as well as long-term neuropsychiatric behaviors such as panic and psychosis. In rodents and monkeys, MDMA is cytotoxic to serotonergic neurons, but this is less clear with humans (3). The onset of its effect can take 20 to 60 minutes to occur, with a peak at 60 to 90 minutes following ingestion. The primary effects last for 3 to 5 hours. MDMA usually induces a relaxed, euphoric state, including emotional openness, empathy, reduction of negative thoughts, and a decrease in inhibitions. Sounds and colors can appear more intense. Accompanying physiological changes can result in severe adverse events (4). The evidence that impaired serotonergic function may be associated with memory deficits in MDMA users is further shown by correlations between alterations in cortical 5-HT2A receptor binding, altered D-fenfluramine-induced cortisol responses, and memory deficits. Reneman et al. have demonstrated higher overall 5-HT2A receptor binding ratios in the brains of an MDMA user group. MDMA-induced 5-HT depletion results in the up-regulation of 5-HT2 receptors. MDMA users show significant deficits in delayed memory tasks, which directly correlates with the increase in 5-HT2A receptor binding ratios. Verkes et al. have observed a significantly reduced cortisol response to D-fenfluramine in MDMA users compared to control subjects. Those who used MDMA also had significantly longer reaction times to visual and auditory stimuli, lower visual recall, and lower working memory scores. The reduced cortisol response correlates significantly with visual recall scores, indicating a significant association between chronic MDMA use, diminished memory performance, and serotonergic neuroendocrine functional deficits (4).

The mechanism of MDMA-induced depletion of the central nervous system (CNS) serotonin (5-hydroxytryptamine, 5-HT) is believed to involve the generation of reactive oxygen species (5). The process of apoptosis is controlled by a diverse range of cell signals, which may originate either extracellularly via extrinsic inducers or intracellularly via intrinsic inducers. Extracellular signals may include toxins, hormones, growth factors, oxides or cytokines, which must either cross the cell membrane or transduce to affect a response. These signals may positively (trigger) or negatively (inhibit) affect apoptosis. A cell initiates intracellular apoptotic signaling in response to a stress or cell suicide. The binding of nuclear receptors by glucocorticoids, heat, radiation, nutrient deprivation, viral infection, hypoxia and increased intracellular calcium concentration (by damage to the membrane) can all trigger the release of intracellular apoptotic signals by a damaged cell. A number of cellular components regulate apoptosis. The Bcl-2 protein is able to inhibit apoptosis by direct action, while Bax and/or Bak promote apoptosis (6). Various stressors and hypothermia protect against d-MDMA-induced neurotoxicitythrough unknown mechanisms (7-10).

Adenosine, a nucleoside produced by cells and tissues in response to a variety of physical and metabolic stresses, mediates physiological activities that include sedation, inhibition of platelet aggregation, and vasodilatation (11). In addition to the dopaminergic pathways, the involvement of neurotransmitter and neuromodulator systems in opioid-dependent mechanisms is documented. The glutamatergic and aminobutyric acid (GABAergic) systems are the most engaged in opioid dependence, although the role of other modulators, such as nitric oxide or adenosine, cannot be ruled out. Endogenous adenosine, a potent inhibitory neuromodulator in the CNS, is known to affect the state of dependence. For example, opioid, ethanol, or benzodiazepine withdrawal signs are attenuated by treatment with adenosine receptors or A2A agonists (12).

A2A is a member of the G protein-coupled receptor family. The A2A receptor is also expressed in the brain, where it plays a role in regulation of glutamate and dopamine release, thus making it a potential therapeutic target for the treatment of conditions such as pain, depression, drug addiction, and Parkinson’s disease. The A2A receptor has some agonists such as glutamyl cysteine synthetase (GCS), in addition to antagonists such as caffeine and (SCH) (13).

2-p-(2-carboxyethyl) phenethylamino-5’-N-ethylcarboxamidoadenosine hydrochloride (CGS 21680) and 5’-N-ethylcarboxamidoadenosine (NECA) inhibit the initiation of cocaine self-administration. According to research, CGS 21680 and NECA inhibit the development of sensitivity to diazepam withdrawal signs, whereas adenosine receptor antagonists intensify diazepam and temazepam withdrawal signs.

The adenosinergic system is well-recognized as an important modulator of dependence, but less is known about the effects of adenosine ligands on the development of hypersensitivity to acute morphine doses that are injected during morphine withdrawal periods. MDMA causes apoptosis in brain tissue and A2A receptors can mediate apoptosis in cells via both known and unknown pathways. The present study is undertaken to investigate the role of the selective adenosine A2A receptor on apoptosis caused by MDMA (11, 12, 14).

## Materials and Methods

### Animals

The experiments were performed on male Sprague Dawley rats (weights: 200–250 g each). Animals were kept three to four per cage at a room temperature of 22 ± 1℃ on natural day-night cycles (Spring). Standard nutrition and tap water were freely available. All the experiments were performed between 9 am and 2 pm.

### Groups

We divided the rats into seven groups. Each group was treated with daily intraperitoneal (IP) injections for a period of one week, as follows: i. MDMA (10 mg/kg); ii. GCS (0.3 mg/kg); iii. SCH (0.3 mg/kg); iv. GCS + SCH (0.3 mg/kg each); v. MDMA (10 mg/kg) + GCS (0.3 mg/kg); vi. MDMA (10 mg/kg) + SCH (0.3 mg/kg); and vii. normal saline (1 cc/kg) as the sham group.

### Surgery

After two weeks injections the animals were euthanized and their striata removed immediately for total RNA and protein extraction, and maintained in N2 in frized at -70℃.

### Western blot analysis

Proteins were extracted by using the RiPA buffer (Gibco, USA) and a cocktail protein inhibitor (Gibco, USA). The concentration of protein was analyzed with Bio-rad protein assay buffer. Protein was run in a 12% polyacrylamid gel and then transferred onto a nitrocellulose membrane after which immunoblotting was performed. We used a primary antibody against Bcl-2 and Bax (Sigma, USA) followed by rat monoclonal secondry antibody for IgG FITC. After the addition of BCIP solution the bands were visualized, scanned, and analyzed with UVItec software version 12.6 (13).

### Reverse transcription PCR

The total RNA from striatum was isolated with TRIzol® (Gibco/Invitrogen, USA) following the manufacturer’s protocol. The PCR reaction mixture 50 µl consisted of 2 µl cDNA in PCR buffer [20 mM tris-HCl (pH= 8.4) and 50 mM KCl] that contained 0.2 mM dNTP mix, 4.5 mM MgCl_2_, 100 ng each of forward and reverse primers, and 1 U Platinum TaqDNA polymerase (Gibco/Invitrogen, USA). sscDNA (cDNA) was synthesized and amplified using specific primers chosen from NCBI for rotin genes.The following primers were used:

Bcl-2
  Forward: 5'-GGT GCC ACC TGT GGT CCA CCT G-3'
  Reverse: 5'-CTT CAC TTG TGG CCC AGA TAG G-3'
Bax
  Forward: 5′-GGT TTC ATC CAG GAT CGA GAC GG-3′
  Reverse: 5′-ACA AAG ATG GTC ACG GTC TGC C-3′
GAPDH
  Forward: 5'-CTG TGT GGA CTT GGG AGA GG-3'
  Reverse: 5'-GGC ATC CAC GAA ACT ACC TT-3'

The PCR product was separated by electrophoresis on 2% agarose gels that contained ethidium bromide (0.15 mg/ml), subsequently visualized with a UV transilluminator UV-tec, Germany, and digitally photographed. The amplicon was quantified densitometrically using Kodak Digital Science software (13).

### Statistical analysis

We repeated all experiments a minimum of three times, from which qualitatively identical results were obtained. Differences between groups were analyzed using the student’s t test or appropriate level of ANOVA. Statistical analyses were performed with the Statistical Package for the Social Sciences (SPSS, version 18). A p<0.05 was considered statistically significant.

### Ethical considerations

The study was approved by the Ethics Committee of Tehran University of Medical Sciences (TUMS).

## Results

**Fig 1:**
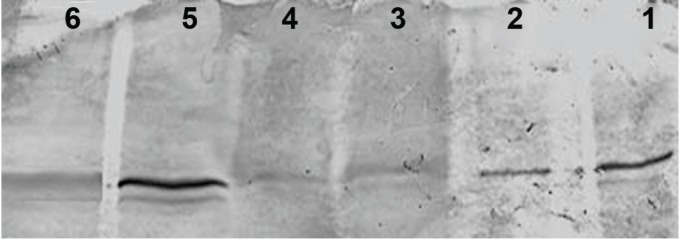
Increase of Bax protein in the MDMA + SCH group. Lane number 1: GCS, 2:MDMA + GCS, 3: Control, 4: MDMA, 5: MDMA + SCH, 6:SCH

**Fig 2:**
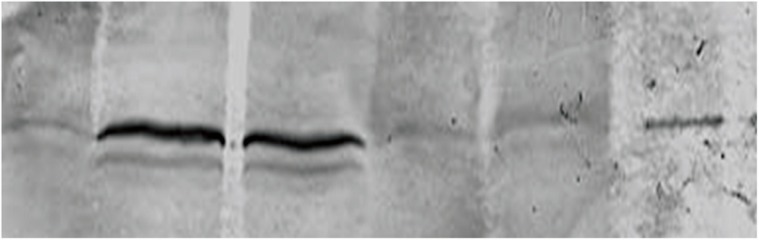
Decreased Bcl-2 protein expression in the MDMA+SCH group. Lane number 1: SCH, 2: MDMA + SCH, 3: Control, 4: MDMA, 5: MDMA + GCS, 6: GCS.

**Fig 3:**
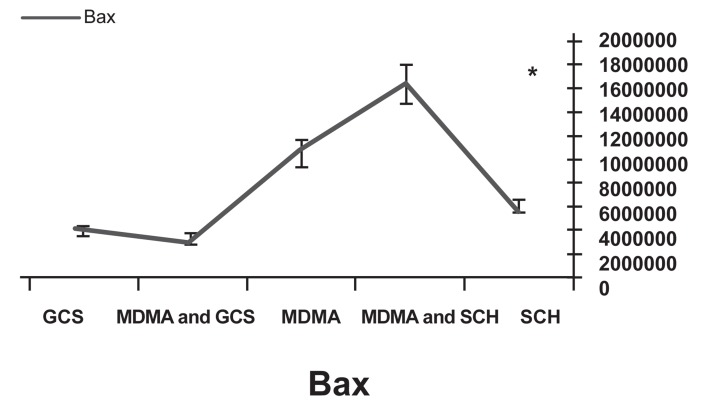
Increased Bax protein in the MDMA + SCH group.

**Fig 4:**
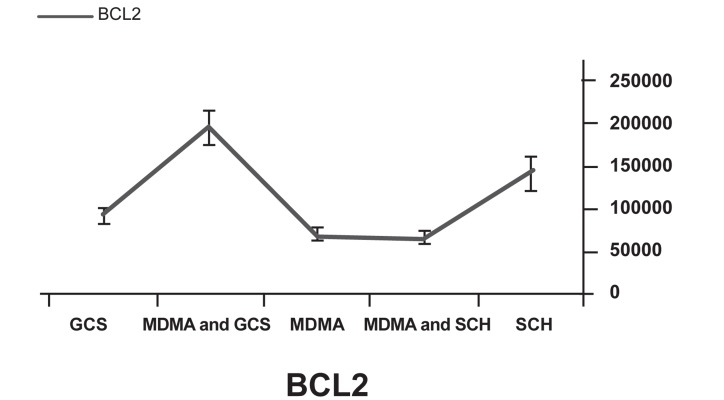
Bcl-2 protein expression in the MDMA+SCH group

**Fig 5:**
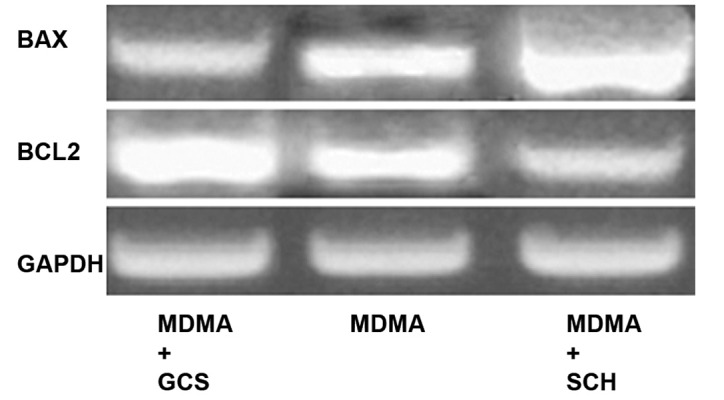
Decreased Bcl-2 mRNA expression in MDMA+SCH group compared to MDMA group.

Western blot analysis showed decreased expression of the Bcl-2 protein in the MDMA+ SCH group compared to the other groups (p<0.05). Bax protein expression in the MDMA + SCH group increased in contrast to the other groups (Figs 1-4). Results of RT-PCR showed a meaningful increase of Bax mRNA expression in the MDMA + SCH group in contrast to the other groups, and decreased Bcl-2 mRNA expression in this same group in contrast to the MDMA group (p<0.05; Fig 5).

## Discussion

Exposure to 15 mg/kg of methamphetamine can produce cell death in the mouse hippocampus after five days (15). A different study shows that exposure of this drug at a dose of 4 mg/kg, four times per day results in cell death (16).

Research shows that cell death is the same as apoptosis (17-19). In this study we have noted that apoptosis plays a considerable role in cell damage caused by MDMA. Some studies show that MDMA can result in cell-to-cell interaction or cell-to-matrix interaction and chronic changes in neurons (20). The deregulations of methamphetamine relate to the production of super oxide and free radicals (21). In animal models, MDMA increases locomotoric actions (22), which occur because of serotonin release (23).

Jayanthi and coworkers have shown that MDMA exposure changed the expression of Bcl-2 in rat brains, increased the expression of Bad, Bax, and Bid, and decreased the expression of Bcl-2 and Bcl-xl (24).

Previous studies have shown that A2A adenosine receptors mediate the neural pathway excited by drugs. In addition to activation, these receptors can result in the release of certain neurotransmitters in the brain such as dopamine and glutamine, which affect behavioral development (25). A2A receptors have an important role in long–term responses to dopaminergic excitatory drugs in rats and monkeys (26). The CGS 21680 A2A agonist receptor reduces sensitization to methamphetamines (27) and GCS decreases the stereotypy of methamphetamines (28).

The supportive role of A2A receptors in the reduction of neural defects due to ischemia is shown by a decrease in apoptosis after the use of A2A receptor agonists that are related to the changes of expression in Bax and Bcl-2 genes (25). We have focused on the role of apoptosis in MDMA cell damage and the corporation of A2A receptor in that. Our results have shown that the use of A2A receptor agonists decreased the effect of MDMA. Thus, the A2A receptor has an important role in the effect of MDMA on brain damage. As a result of the analysis of Bax and Bcl-2 proteins, this role has been determined to be related to apoptosis. We showed this mechanism occurred via a change in Bax and Bcl-2 expression. We also demonstrated that exposure to 10 mg/kg MDMA after one week increased Bax protein expression and caused a decrease in Bcl-2 protein expression. Our results showed that the use of GCS can reduce side effects of MDMA by causing increased Bcl-2 expression and decreased Bax expression. Our results agreed with those of other studies. Additional studies should be performed to test the other effects of GCS in the brain and confirm the use of it to decrease MDMA effects.

## Conclusion

The use of A2A receptor agonists in this drug can reduce some of the side effects of MDMA.

## References

[1] Capela JP, Carmo H, Remião F, Bastos ML, Meisel A, Carvalho F (2009). Molecular and cellular mechanisms of ecstasy-induced neurotoxicity: an overview. Mol Neurobiol.

[2] Riezzo I, Cerretani D, Fiore C, Bello S, Centini F, D'Errico S (2010). Enzymatic-nonenzymatic cellular antioxidant defense systems response and immunohistochemical detection of MDMA, VMAT2, HSP70, and apoptosis as biomarkers for MDMA (Ecstasy) neurotoxicity. J Neurosci Res.

[3] Simantov R, Tauber M (1997). The abused drug MDMA (Ecstasy) induces programmed death of human serotonergic cells. FASEB J.

[4] Green AR, Mechan AO, Elliott JM, O’shea E, Colado MI (2003). The pharmacology and clinical pharmacology of 3, 4-methylenedioxymethamphetamine (MDMA, "ecstasy"). Pharmacol Rev.

[5] Shankaran M, Yamamoto BK, Gudelsky GA (1999). Involvement of the serotonin transporter in the formation of hydroxyl radicals induced by 3, 4-methylenedioxymethamphetamine. Eur J Pharmacol.

[6] Sheikh MS, Huang Y (2003). Death receptor activation complexes: it takes two to activate TNF receptor 1. Cell Cycle.

[7] Johnson EA, O'Callaghan JP, Miller DB (2004). Brain concentrations of d-MDMA are increased after stress. Psychopharmacology (Berl).

[8] Broening HW, Bowyer JF, Slikker W Jr (1995). Age-dependent sensitivity of rats to the long-term effects of the serotonergic neurotoxicant (+/-)-3, 4-methylenedioxymethamphetamine (MDMA) correlates with the magnitude of the MDMA-induced thermal response. J Pharmacol Exp Ther.

[9] Blessing WW, Seaman B (2003). 5-hydroxytryptamine(2A) receptors regulate sympathetic nerves constricting the cutaneous vascular bed in rabbits and rats. Neuroscience.

[10] Saadat KS, Elliott JM, Colado MI, Green AR (2004). Hyperthermic and neurotoxic effect of 3,4-methylenedioxymethamphetamine (MDMA) in guinea pigs. Psychopharmacology (Berl).

[11] Kaplan GB, Bharmal NH, Leite-Morris KA, Adams WR (1999). Role of adenosine A1 and A2A receptors in the alcohol withdrawal syndrome. Alcohol.

[12] Listos J, Talarek S, Fidecka S (2008). Involvement of adenosine receptor agonists on the development of hypersensitivity to acute dose of morphine during morphine withdrawal period. Pharmacol Rep.

[13] Katebi M, Soleimani M, Cronstein BN (2009). Adenosine A2A receptors play an active role in mouse bone marrow-derived mesenchymal stem cell development. J Leukoc Biol.

[14] Shimazoe T, Yoshimatsu A, Kawashimo A, Watanabe S (2000). Roles of Adenosine A (1) and A (2A) receptors in the expression and development of methamphetamine-induced sensitization. Eur J Pharmacol.

[15] Schmued LC, Bowyer JF (1997). Methamphetamine exposure can produce neuronal degeneration in mouse hippocampal remnants. Brain Res.

[16] Eisch AJ, Marshall JF (1998). Methamphetamine neurotoxicity: dissociation of striatal dopamine terminal damage from parietal cortical cell body injury. Synapse.

[17] Deng X, Cadet JL (2000). Methamphetamine-induced apoptosis is attenuated in the striata of copper-zinc superoxide dismutase transgenic mice. Brain Res Mol Brain Res.

[18] Deng X, Jayanthi S, Ladenheim B, Krasnova IN, Cadet JL (2002). Mice with partial deficiency of c-Jun show attenuation of methamphetamine-induced neuronal apoptosis. Mol Pharmacol.

[19] Deng X, Wang Y, Chou J, Cadet JL (2001). Methamphetamine causes widespread apoptosis in the mouse brain: evidence from using an improved TUNEL histochemical method. Brain Res Mol Brain Res.

[20] Thiriet N, Ladenheim B, McCoy MT, Cadet JL (2002). Analysis of ecstasy (MDMA)-induced transcriptional responses in the rat cortex. FASEB J.

[21] Schmued LC, Bowyer JF (1997). Methamphetamine exposure can produce neuronal degeneration in mouse hippocampal remnants. Brain Res.

[22] Dafters RI (1994). Effect of ambient temperature on hyperthermia and hyperkinesis induced by 3, 4-methylenedioxymethamphetamine (MDMA or "ecstasy") in rats. Psychopharmacology (Berl).

[23] Liechti ME, Baumann C, Gamma A, Vollenweider FX (2000). Acute psychological effects of 3,4-methylenedioxymethamphetamine (MDMA, "Ecstasy") are attenuated by the serotonin uptake inhibitor citalopram. Neuropsychopharmacology.

[24] Jayanthi S, Deng X, Bordelon M, McCoy MT, Cadet JL (2001). Methamphetamine causes differential regulation of pro-death and anti-death Bcl-2 genes in the mouse neocortex. FASEB J.

[25] Rosin DL, Hettinger BD, Lee A, Linden J (2003). Anatomy of adenosine A2A receptors in brain: morphological substrates for integration of striatal function. Neurology.

[26] Bibbiani F, Oh JD, Petzer JP, Castagnoli N Jr, Chen JF, Schwarzschild MA (2003). A2A antagonist prevents dopamine agonist-induced motor complications in animal models of Parkinson’s disease. Exp Neural.

[27] Shimazoe T, Yoshimatsu A, Kawashimo A, Watanabe S, Watanabe S (2000). Roles of adenosine A(1) and A(2A) receptors in the expression and development of methamphetamine-induced sensitization. Eur J Pharmacol.

[28] Poleszak E, Malec D (2000). Influence of adenosine receptor agonists and antagonists on amphetamine-induced stereotypy in rats. Pol J Pharmacol.

